# *Winogradskyella luteola *sp.nov., *Erythrobacter ani* sp. nov., and *Erythrobacter crassostrea* sp.nov., isolated from the hemolymph of the Pacific Oyster *Crassostrea gigas*

**DOI:** 10.1007/s00203-022-03099-y

**Published:** 2022-07-14

**Authors:** Hani Pira, Chandra Risdian, Mathias Müsken, Peter J. Schupp, Joachim Wink

**Affiliations:** 1 Helmholtz Centre for Infection Research (HZI), Microbial Strain Collection (MISG), 38124 Brunswick, Germany; 2 Central Facility for Microscopy, Helmholtz Centre for Infection Research (HZI), 38124 Brunswick, Germany; 3 Environmental Biochemistry, Institute for Chemistry and Biology of the Marine Environment (ICBM), Oldenburg, Germany; 4 Helmholtz Institute for Functional Marine Biodiversity at the University of Oldenburg, Ammerländer Heerstraße 231, 26129 Oldenburg, Germany; 5 Research Center for Environmental and Clean Technology, National Research and Innovation Agency (BRIN), Bandung, 40135 Indonesia

**Keywords:** Bioremediation, *Erythrobacter*, Polyphasic taxonomy, *Winogradskyella*, Genome mining

## Abstract

Three new bacterial strains, WHY3^T^, WH131^T^, and WH158^T^, were isolated and described from the hemolymph of the Pacific oyster *Crassostrea gigas* utilizing polyphasic taxonomic techniques. The 16S rRNA gene sequence analysis revealed that strain WHY3^T^ was a member of the genus *Winogradskyella*, whereas strains WHI31^T^ and WH158^T^ were members of the genus *Erythrobacter*. According to the polygenomic study the three strains formed individual lineages with strong bootstrap support. The comparison of dDDH-and ANI values, percentage of conserved proteins (POCP), and average amino acid identity (AAl) between the three strains and their relatives established that the three strains represented two separate genera. Menaquinone-6 was reported as the major respiratory quinone in strain WHY3^T^ and Ubiquinone-10 for strains WH131^T^ and WH158^T^, respectively. The major cellular fatty acids for strain WHY3^T^ were C_15:0_, anteiso-C_15:1_ ω7c, iso-C_15:0_, C_16:1_ω7c. The major cellular fatty acids for strains WH131^T^ and WH158^T^ were C_14:0_2-OH and t_18:1_ω12 for WH131^T^ and C_17:0,_ and C_18:1_ω7c for strain WH158^T^. Positive Sudan Black B staining Indicated the presence of polyhydroxyalkanoic acid granules for strains WH131^T^ and WH158^T^ but not for strain WHY3^T^. The DNA G + C contents of strains WHY3^T^, WH131^T^ and WH158^T^ were 34.4, 59.7 and 56.6%, respectively. Gene clusters predicted some important genes involved in the bioremediation process. Due to the accomplishment of polyphasic taxonomy, we propose three novel species *Winogradskyella luteola* sp.nov. (type strain WHY3^T^ = DSM 111804^T^ = NCCB 100833^T^), *Erythrobacter ani* sp.nov. (WH131^T^ = DSM 112099^T^ = NCCB 100824^T^) and *Erythrobacter crassostrea* sp.nov. (WH158^T^ = DSM 112102^T^ = NCCB 100877^T^).

**Supplementary Information:**

The online version contains supplementary material available at 10.1007/s00203-022-03099-y.

## Introduction

Pacific oyster *Crassostrea gigas* is the most globally diverse in various environments. This kind of Oyster currently produces more than any other aqua product in the world. The Pacific oyster has the ability to significantly modify its habitat, benefiting and harming native species and ecosystems alike. It is an important invasive aquaculture species that has the potential to outcompete native species and as a habitat-forming species to function as a stepping stone for other non-native species (e.g., the brown algae *Sargassum muticum* in the North Sea).

Pacific oysters are ecosystem engineers, having a significant physical influence on their habitats (Dumbauld et al. 2009). *C. gigas* forms thick mats known as oyster reefs, and when they reach a certain density, they trigger physical changes in the surrounding environment. Oysters are well-known for their capacity to transform soft substrates such as mud and silt into hard substrates. Numerous studies of oyster beds in various places have shown larger concentrations of benthic invertebrates, such as crabs, bivalves, and worms, living on the oyster beds' hard substrate compared to the surrounding soft substrate (Dumbauld et al. 2009). They also provide a hard substrate for various macroalgae to grow on. Besides altering the benthic environment, oysters are filter feeders, consuming suspended plankton and organic debris. It has been a source of great interest for the study of associated marine bacteria due to their accumulation in various tissues such as the gills, gastrointestinal tissues, and mantle (Li et al. 2017), as well as in the circulatory fluid system, the hemolymph, which has been targeted as the most promising component to study oyster microbiota due to its role in the immune system (Li et al. 2017).

Nedashkovskaya et al. identified the genus *Winogradskyella*, a member of the family *Flavobacteriaceae* in the phylum Bacteroidetes (He et al. 2019). The genus *Winogradskyella* has 44 species with validly published names. Following several revisions of the genus description, the genus is now defined as catalase-positive, strictly aerobic or facultatively anaerobic, motile by gliding, yellowish to orange-colored, rod- or cocci-shaped bacteria that contain phosphatidylethanolamine as a major polar lipid and menaquinone-6 (MK-6) as a major respiratory quinone (Song et al. 2018).

Shiba and Simidu erected the bacterium genus *Erythrobacter* under the family *Erythrobacteraceae* (Lee et al. 2005) with the identification of a single type species of *Erythrobacter longus* (Shiba et al. 1982). *Erythrobacter* is currently comprised of 12 species with validly published names (Xu et al. 2020). They are abundant in marine habitats and have been isolated and identified largely from tidal fat deposits, marine cyanobacterial mats, and marine plants (Shiba and Simidu 1982; Park et al. 2020). Significant features of *Erythrobacter* species include negative Gram staining, aerobic or facultatively anaerobic chemoorganotrophic metabolisms, and the synthesis of ubiquinone-10 as their predominant isoprenoid quinone (Tonon et al. 2014).

The bioremediation procedure indicates the use of microorganisms' metabolic ability to clean up polluted environments. It refers to microorganisms' metabolic capacity to mineralize or change organic pollutants into less hazardous chemicals that may be incorporated into natural biogeochemical cycles. Marine bacteria are organisms that are naturally exposed to harsh environments. Therefore, marine bacteria with bioremediation capacity might be great candidates for the biological treatment of harsh contaminated ecosystems. For instance, copper becomes toxic in higher concentrations (for example, > 0.08 µM Cu), impairing the metabolic activities of marine organisms. Bioremediation is a cost-effective method of treating polluted environments. Copper sorption by highly efficient bacteria may be employed to remove copper from polluted locations (Leal et al. 2018).

In this study, we isolated and performed the polyphasic taxonomy of three novel species belonging to the *Erythrobacter*, and *Winogradskyella* genus (Strains WHY3^T^, WH131^T^and WH158^T^) isolated from the hemolymph of the Pacific oyster *Crassostrea gigas* and, based on genome data, predicted gene clusters for bioremediation and other important processes like polyhydroxyalkanoic acid.

## Materials and methods

### Isolation

The samples were collected of wild oysters from the Wadden Sea near Wilhelmshaven, Germany (Latitude: 53.5131, Longitude: 08.14714) in December 2019, both valves were cleaned externally with a brush and sterile water to eliminate any dirt or debris that may contaminate the extraction process. The adductor muscle was entirely excised with a scalpel blade, and the remainder of the tissues were pooled together. The adductor muscle can be used to capture the hemolymph contained within (King et al. 2019). Serial dilutions of samples were performed (1:10) to a 10^–5^ dilution using 100 µL of sample and 900 µL sterile water in 1.5 mL Eppendorf tubes. Artificial saltwater medium (ASW) supplemented with vitamin, and antifungal agent (ATI Coral Ocean salt (39 g/L), agar (15 g/L), nicotinic acid (20 mg/L), thiamine (vitamin B1, 10 mg/L), biotin (vitamin B7, 2 mg/L), 4-aminobenzoic acid (10 mg/L), pantothenic acid (5 mg/L), pyridoxamine (vitamin B6, 50 mg/L), cyanocobalamin (vitamin B12, 20 mg/L), and cycloheximide (100 mg/L), pH 7.3) was used to perform preliminary isolation. The incubation was performed for 6 days at 30 ℃.

The orange and yellow colonies (strains WHY3^T^, WH131^T^, and WH158^T^) were selected and transferred to Bacto marine agar (MA, Difco 2216), where they were purified successively by streaking over the same medium. The strains were held at − 80 ℃ for long-term preservation.

## Morphological, physiological and biochemical studies

Cells were grown on MB (Difco marine broth 2216) media for 3 days at 30 °C followed by morphological observations, including motility, using a light microscope (Zeiss Axio Sc pie. A1 microscope). Cells grown in MB media for 2 days at 30 °C were fixed with aldehydes (final concentrations: 5% formaldehyde and 2% glutaraldehyde), dehydrated in a gradient series of acetone, critical point dried, and coated with gold–palladium according to a previously published protocol (Landwehr et al. 2018). Images were acquired at various magnifications using a Zeiss Merlin field emission scanning electron microscope (FESEM) equipped with a 25:75% ratio of Everhart–Thornley SE and Inlens SEM detectors. The sodium chloride tolerance of strains WHY3^T^, WH131^T^, and WH158^T^ was determined using the following NaCl (w/v) concentrations: 0%, 2.5%, 5.0%, 7.5%, 10%, 15%, 25%, and 30% using the method of Kutzner (1981). The growth of bacteria on solitary carbon and nitrogen sources was evaluated using Microlog GEN III plates (Biolog). Sudan black B staining was employed at a concentration of 3% (w/v in 70% ethanol) to identify polyhydroxyalkanoate granules (PHAs) (Legat et al. 2010). MA medium was used to assess growth over a variety of temperatures (4, 15, 20, 25, 30, 35, 40, and 45 °C) and pH values (pH 5, 6, 7, 8, 9, 10, and 11). Antibiotic susceptibility tests were performed on MA medium over 48 h with the following antibiotics: gentamycin (10 µg/mL), oxytetracycline (10 µg/mL), thiostrepton (50 µg/mL), trimethoprim (50 µg/mL), ampicillin (10 µg/mL), chloramphenicol (30 µg/mL), polymyxin (50 µg/ml), spectinomycin (50 µg/mL), kanamycin (30 µg/mL), cephalosporin (50 µg/mL), fusidic acid (50 µg/mL), bacitracin (50 µg/mL), erythromycin (15 µg/mL), and tetracycline (50 µg/mL).Table S1. Api Zym (Humble et al. 1977), Api 20NE, Api 20E (bioMe'rieux) (O'Hara et al. 1992), and GEN III microplate (Biolog) were used in the biochemical investigation.

### 16S rRNA gene analysis

The Invisorb Spin Plant Mini Kit was used to extract genomic DNA in accordance with the manufacturer's instructions (Stratec Molecular, Germany).PCR amplification of the 16S rRNA gene was applied with the primer F27 (5′–AGAGTTTGATCMTGGCTCAG–3′) and 1492R (5′–TACGGYTACCTTGTTACGACTT–3′) (Chaiya et al. 2019). The 16S rRNA gene was sequenced employing an Applied Biosystems 3730XL automated sequencer (ABI). BioEdit software was used to modify and assemble the sequence (version 7.0.5.3) (Hall 1999). The 16S rRNA gene sequence of strains WHY3^T^ (1343 bp), WH131^T^ (1443 bp), and WH158^T^ (1486 bp) were almost completely sequenced and submitted to GenBank under the accession number MW888983, MW888981, and MW888982, respectively. The phylogenetically closest strains of WHY3^T^, WH131^T^, and WH158^T^ were determined based on 16S rRNA gene sequence similarity using the EZBioCloud system (https://www.ezbiocloud.net/) (Yoon et al. 2017a). Phylogenetic analysis of the 16S rRNA gene of strains WHY3^T^ was inferred using the GGDC web server for closely similar type strains (http://ggdc.dsmz.de/) (Meier-Kolthoff et al. 2013). The sequence was analyzed using a single-gene adaptation of the DSMZ phylogenomics program (Meier-Kolthoff et al. 2014). Multiple sequence alignment was performed using MUSCLE (Edgar 2004). Randomized Axelerated Maximum Likelihood (RAxML) (Stamatakis 2014) and TNT (Tree analysis using New Technology) (Goloboff et al. 2008) programs were applied to estimate Maximum likelihood (ML) and Maximum parsimony (MP) trees, respectively. We employed rapid bootstrapping using the autoMRE (extended majority rule) bootstrapping parameters(Pattengale et al. 2010). 1000 bootstrapping replicates, tree bisection, reconnection branch switching, and ten random sequence addition repetitions were used in the case of MP. The X2 tests used in PAUP* (Phylogenetic Analysis using Parsimony*) were used to analyze the sequences (Swofford and Sullivan 2003). The twelve valid *Erythrobacter* sequences were aligned using Clustal X for strains WH131^T^ and WH158^T^, and phylogenetic trees were constructed using the maximum likelihood algorithms of MEGA X software (Kumar et al. 2018). The phylogenetic trees were supported by bootstrap for the resampling test with 100 replicates for the maximum likelihood algorithms.

## Chemotaxonomy

Bacterial biomass was produced and collected after 7 days at 30 °C in a 250 mL flask containing 100 mL MB medium on a rotary shaker (160 revolutions per minute). The chemotaxonomic study was performed from freeze-dried biomass. Isoprenoid quinone production and analysis were conducted according to Minnikin (1984). Compounds were evaluated using high-performance liquid chromatography coupled with the diode array detector and mass spectrometer (HPLC-DAD-MS). The mobile phase consisted of 35% isopropanol + 1% water + 0.1% formic (solvent A) and 65% acetonitrile + 1% water + 0.1% formic acid (solvent B) and were used at a flow rate of 0.3 mL/min under isocratic conditions. Isoprenoid quinones were separated using a Waters ACQUITY UPLC BEH C18 column (2.1 × 50 mm, 1.7 m). Fatty acid extraction and methylation were carried out according to Sasser's procedure (Sasser 1990). Fatty acid methyl esters (FAME) were analyzed utilizing a gas chromatograph equipped with a flame ionization detector from Agilent (FID). Using a Macherey Nagel Optima 5 column, the methyl esters of fatty acids were extracted (5%phenyl, 95% dimethylpolysiloxane; 50 m length; 0.32 mm inner diameter; 0.25 m film thickness). Their retention periods were compared to standards (in-house reference standard) to identify specific fatty acid methyl esters.

### Whole-genome analysis

Illumina's next-generation sequencing technology using MiSeq 600 cycle v3 was employed for whole-genome sequencing, and Unicycler was applied for genome de novo assembly (Wick et al. 2017). To estimate the purity of the 16S rRNA gene in whole-genome data, the ContEst16S technique was utilized (https://www.ezbiocloud.net/tools/contest16s) (Lee et al. 2017a). To accomplish automated genome annotation, the NCBI Prokaryotic Genome Annotation Pipeline (PGAP) was employed (Tatusova et al. 2016). In addition, the draft genome assembly was submitted to the RAST (Rapid Annotation Using Subsystem Technology) database for metabolic reconstruction study (https://rast.nmpdr.org/) (Aziz et al. 2008). A phylogenomic tree was generated using the whole-genome sequence of strains WHY3^T^, WH131^T^, and WH158^T^ and their closest phylogenetic relatives using the Type (Strain) Genome Server (TYGS) (Meier-Kolthoff et al. 2022). The Ezbiocloud and NCBI databases were used to extract whole-genome sequences of *Winogradskyella flava* KCTC 52348^T^*, Winogradskyella ouciana* ZXX205^T^*, Winogradskyella echinorum* KCTC 22026^T^*, Erythrobacter insulae* JBTF-M21^T^*, Erythrobacter rubeus* KMU-140^T^*, Erythrobacter longus* DSM 6997^T^*, Erythrobacter litoralis* DSM8509^T^, *Pseudopontixanthobacter vadosimaris* JL3514^T^ and *Parerythrobacter jejuensis* JCM 16677^T^. Strains WHY3^T^, WH131^T^, and WH158^T^ genome sequences were submitted to the Type (Strain) Genome Server (TYGS) (https://tygs.dsmz.de; accessed on 05 March 2022). All pairwise comparisons for phylogenomic inference were performed using Genome BLAST Distance Phylogeny (GBDP), and precise intergenomic distances were obtained using the 'trimming' process and distance formula d5 (Meier-Kolthoff et al. 2013). One hundred distance replicates were considered each. Using the specified settings, we created digital DDH (dDDH) values and confidence intervals using the Genome-to-Genome Distance Calculator (GGDC 3.0) (Meier-Kolthoff et al. 2022). Intergenomic distances and branch support from FASTME 2.1.6.1 were used to build a balanced minimal evolution tree, which included subtree pruning and regrafting postprocessing (SPR) (Lefort et al. 2015). The tree's branch support was calculated using 100 pseudobootstrap replications. The average nucleotide identity (ANI), genome size, and guanine and cytosine (G + C) content were determined using the OrthoANIu method (Yoon et al. 2017b) (https://www.ezbiocloud.net/tools/ani). To support the classification of strains WH131^T^ and WH158^T^ in the appropriate genus, the average amino acid identity (AAI) value (http://enve-omics.ce.gatech.edu/aai/index) and the percentage of conserved proteins (POCP) value (https://github.com/2015qyliang/POCP) were calculated (Qin et al. 2014). The RAST algorithm v1.073 from the KBase database https://narrative.kbase.us/ genes was used in the field of genome mining (The genome features were functionally annotated using the following algorithms: Kmers V2; Kmers V1; and protein similarity). The most important genes involving bioremediation from isolated strains of the genus *Winogradskyella* and *Erythrobacter* were annotated as follows: the gene responsible for converting the toxic form of mercury to the nontoxic form and uptake of mercury via membranes which carried the Hg(II) into the cytoplasm (*mer*A, *mer*B, *Mer*C, *Mer*E, *Mer*F, *Mer*T, *Mer*P) (Sone et al. 2013; Zhang et al. 2020); polyaromatic hydrocarbons (PAHs) are abundant in nature and are a significant source of environmental concern owing to their persistence, toxicity, mutagenicity, and carcinogenicity (Cerniglia 1993); anthracene and phenanthrene may be oxidized by the *Phn*A dioxygenase as carbon and energy source (Kasai et al. 2003); polyhydroxyalkanoic acid (PHAs) are stored inside cells as carbon and energy reserves (Takahashi et al. 2017); degradation of phenol (*dmp*N) (Selvaratnam et al. 1997); chromate resistance genes (*chr*B)(Aguilar-Barajas et al. 2008); heavy metal resistance protein cobalt–zinc–cadmium (*czc*A*, czc*D) (Abdelatey et al. 2011); nickel–cobalt–cadmium resistance protein (*ncc*A, *ncc*X) (Lee et al. 2019); copper resistance (*cus*A, *cus*B, *cus*C, *cus*F, Copper resistance protein B) (see Table 3 for details) (Bondarczuk and Piotrowska-Seget 2013).

The draft genome of strains WHY3^T^, WH131^T^, and WH158^T^ was submitted to NCBI/GenBank with the accession number (JAGSPD000000000), (JAGSPB000000000), and (JAGSPC000000000), respectively.

### Antimicrobial activity

Strains WHY3^T^, WH131^T^, and WH158^T^ were grown for 5 days at 30 ℃ on a shaker in 250-ml Erlenmeyer flasks that contained 100 mL of MB medium with 2% (v/v) XAD-2 polymeric resin (160 revolutions per minute). The separation of XAD-2 was accomplished by separating the resin with a paper filter from the media. Acetone was used to prepare the crude extract from the XAD-2. A rotary evaporator was used to dry the extract at a temperature of 40 °C. The dried extract was diluted in 1 mL methanol and evaluated for antimicrobial activity against a variety of the following bacteria: *Citrobacter freundii* DSM 30,039, *Staphylococcus aureus* Newman*, Escherichia coli* wild type BW25113, *Escherichia coli* acrB JW25113, *Mycobacterium smegmatis* ATCC 700,084, *Pseudomonas aeruginosa* DSM 19,882, *Acinetobacter baumannii* DSM 30,008, *Bacillus subtilis* DSM 10, *Mucor hiemalis* DSM 2656, *Wickerhamomyces anomalus* DSM 6766, and *Candida albicans* DSM 1665. The serial dilution procedure was applied using 96-well microplates in accordance with Khosravi Babadi et al. (2021).

## Result and discussion

### Morphological, physiological and biochemical results

Cell size measurements are described in the description part for each strain. The electron microscopy images are available in supplementary file (Fig. S1).

The ideal temperature for growth for all three strains was 30 °C. Besides pH ranges, the tolerance of sodium chloride was recorded; the results of the biochemical property-based Api ZYM, Api 20NE, and Api 20E tests indicated positive activity for all strains WHY3^T^, WH131^T^, and WH158^T^ and related closes type strains for catalase, oxidase, phosphatase alkaline, leucin arylamidase, valine arylamidase; and negative for α-galactosidase, β-glucuronidase, N-acetyl-β-glucosaminidase, α-mannosidase, α-fucosidase. Other comparisons for phenotypic characteristics and also Biolog Gen III system results between the isolated strains and closet-related strains are observable in Table 1 and description. Sudan Black B indicates a preference for PHAs granules for strains WH131^T^ and WH158^T^ but not for WHY3^T^.

**Table 1:** Comparison of phenotypic characteristics that distinguish strains WHY3^T^, WH131^T^, and WH158^T^ from the most closely related type strains

**Genus name** **Species name**	***Winogradskyella***					***Erythrobacter***				
	**1**	**2**	**3**	**4**	**5**	**6**	**7**	**8**	**9**	**10**
Color of colony	Yellow	Yellow	Yellow	Yellow	Yellowish orange	Orange	strong orange	Reddish orange	Orange	Orange Or Red
Temperature range for growth (°C)	5–40	10–45*	4–45^†^	4–37^↓^	20–35	20–35	30(opt)^‡^	10–45**	15–40	10–40
pH spectrum for growth	6–9	7–9*	6–9^†^	ND	6–9	6–8	6–8^‡^	6–9.5**	6–9	6–8
NaCl (optimum) for growth (%)	2.5	2*	3^†^	1–6^↓^	2.5	2.5	1–2^‡^	2**	2.5	0
ApiZym										
Esterase (C4)	+	+*	+^†^	+^↓^	+	+	+^‡^	–**	+	+
Esterase lipase (C8)	+	+*	+^†^	+^↓^	+	+	+^‡^	–**	+	+
Lipase (C14)	–	+*	–^†^	–^↓^	–	+	–^‡^	–**	W	–
Cystine arylamidase	–	–*	+^†^	+^↓^	+	+	–^‡^	–**	+	W
Trypsin	–	+*	+^†^	–^↓^	+	+	+^‡^	+**	+	–
α-Chymotrypsin	–	+*	–^†^	+^↓^	+	+	+^‡^	+**	+	–
Phosphatase acid	+	+*	+^†^	+^↓^	+	+	–^‡^	+**	+	–
Naphtol-AS-BI-phosphohydrolase	+	+*	+^†^	+^↓^	W	+	+^‡^	+**	+	W
β-Galactosidase	–	–*	–^†^	+^↓^	–	–	+^‡^	+**	–	–
α-Glucosidase	–	–*	–^†^	–^↓^	–	–	+^‡^	–**	–	–
β-Glucosidase	–	W*	–^†^	–^↓^	–	–	–^‡^	–**	–	–
Api 20NE-Api 20E										
Nitrate reduction	–	–*	–^†^	–^↓^	+	–	–^‡^	+**	+	–
Indole	–	–*	ND	–^↓^	–	–	ND	–**	+	ND
Arginine dihydrolase	–	–*	ND	ND	+	+	ND	–**	+	ND
Lysin decarboxylase	–	ND	ND	ND	–	–	ND	–**	–	ND
Urease	–	–*	ND	–^↓^	–	–	–^‡^	–**	+	–
Aesculin hedrolysis	–	+*	ND	W^↓^	+	+	–^‡^	ND**	+	+
Gelatin hedrolysis	–	+*	+^†^	+^↓^	–	–	+^‡^	+**	+	
Tween 80 hydrolysis	–	+*	+^†^	–^↓^	+	–	+^‡^	+**	+	+
Tween 40 hydrolysis	–	ND	+^†^	–^↓^	+	+	ND	ND**	ND	ND
Tween 20 hydrolysis	ND	–*	–^†^	–^↓^	ND	ND	ND	ND**	ND	ND
Ornithine decarboxylase	–	ND	ND	ND	–	–	ND	–**	–	–
H2S production	–	–*	ND	–^↓^	–	–	ND	–**	–	–
Acetoin	+	ND	ND	–^↓^	–	–	ND	ND**	–	–
Assimilation of:										
d-glucose	+	–*	ND	+^↓^	+	+	+^‡^	+**	+	+
l-arabinose	–	–*	ND	–^↓^	–	–	–^‡^	ND**	+	–
d-mannose	–	–*	ND	+^↓^	+	+	–^‡^	+**	–	–
d-mannitol	–	–*	ND	–^↓^	+	+	ND	+**	+	–
*N*-acetyl-glucosamine	–	–*	ND	–^↓^	–	–	ND	+**	–	–
d-maltose	–	–*	ND	–^↓^	+	+	–^‡^	+**	+	–
Potassium gluconate	–	–*	ND	–^↓^	–	–	ND	ND**	–	–
Capric acid	–	–*	ND	–^↓^	–	–	ND	ND**	–	–
Adipic acid	–	–*	ND	–^↓^	–	–	ND	ND**	–	–
Malic acid	+	–*	ND	–^↓^	+	–	ND	+**	–	–
Trisodium citrate	–	–*	ND	–^↓^	–	–	–^‡^	ND**	+	–
Phenylacetic acid	–	–*	ND	ND	+	+	ND	ND**	–	–
Citrate	–	ND	ND	–	–	–	–^‡^	–**	+	–
Fermentation of:										
d-glucose	–	–*	ND	–^↓^	–	–	ND	ND**	+	+
d-mannitol	–	–*	+^†^	–^↓^	–	–	ND	ND**	+	–
Inositol	–	ND	ND	–^↓^	–	–	ND	ND**	–	–
d-sorbitol	–	ND	ND	–^↓^	–	–	ND	ND**	+	–
l-rhamnose	–	–*	+^†^	–^↓^	–	–	ND	ND**	–	–
d-sucrose	–	–*	+^†^	–^↓^	–	–	ND	ND**	+	–
d-melibiose	–	ND	ND	–^↓^	–	–	ND	ND**	+	–
Amygdalin	–	ND	+^†^	ND	–	–	ND	ND**	–	–
l-arabinose	–	ND	+^†^	–^↓^	–	–	ND	ND**	+	–
Susceptibility to:										
Gentamycin	–	–*	ND	–^↓^	+	+	–^‡^	ND	+	–
Ampicillin	–	+*	ND	–^↓^	–	–	–^‡^	ND	–	–
Kanamycin	–	–*	ND	–^↓^	+	+	–^‡^	ND	+	+
Vancomycin	+	+*	–^†^	ND	–	–	ND	–**	–	–
Lincomycin	+	ND	–^†^	+^↓^	–	–	–^‡^	–**	–	–
Polar lipids	PE, GL 6AL, 4L	PE, 4AL 2L	PE, 4GL 4AL, 3L	PE^↓^	DPG, PE PG, SGL 2PC,4L 2AL,2GL	DPG, PE PG, SGL PC,4L 2AL,GL	DPG, PE PG, SGL PC, 4L 1GL^‡^	PE, PG PL, 2L**	DPG, PE PG, SGL PC,2L AL,GL	DPG, PE PG, SGL PC,5L 2AL,
Major fatty acid	C_15:0_, anteiso-C_15:1_ ω7c, iso-C_15:0_, C_16:1_ω7c, unknown	iso-C_15:0_, iso-C_15:1_ G unknown	iso-C_15:0_ iso-C_15:1_ G iso-C_16:0_ iso-C_16:0_ 3-OH	iso-C_15: 1_, iso-C_15: 0_, C_15: 0_, iso-C_15: 0_ 3-OH, iso-C_17: 0_ 3-OH	C_14:0_2-OH t_18:1_ω12 Unknown	C_17:0_ C_18:1_ω7c Unknown	C_17:1_ω6c C_18:1_ω7c	C_16:0_ iso-C_18:0_ C_18:1_ω7c	anteiso-C_17:0_ t_18:1_ω12 C_14:0_2-OH Unknown	C_16:0_ C_16:1_ω7c t_18:1_ω12
G+C content (%)	34.4	34.8	35.5	32.3	59.7	56.6	57.0	60.6	57.4	65.2
Contigs	51	11	341	10	7	7	3	4	14	22
No. of protein	3194	3362	3179	3284	3011	2447	2770	2901	3250	3050
rRNA	3	3	10	3	3	3	3	3	3	3
tRNA	48	41	36	40	44	41	41	43	42	46
No. of Gene	3276	3453	3349	3375	3077	2506	2823	2987	3325	3142
Other RNA	4	4	5	4	4	2	3	4	3	3
Pseudogene	27	43	119	44	15	13	6	36	27	40

Strains: 1, *Winogradskyella luteola* WHY3^T^; 2, *Winogradskyella flava* KCTC 52348^T^; 3, *Winogradskyella ouciana* ZXX205^T^; 4, *Winogradskyella echinorum* KCTC 22026^T^; 5, *Erythrobacter ani* WH131^T^; 6, *Erythrobacter crassostrea* WH158^T^; 7, *Erythrobacter insulae* JBTF-M21^T^; 8, *Erythrobacter rubeus* KMU-140^T^; 9, *Erythrobacter longus* DSM 6997^T^; 10, *Erythrobacter litoralis* DSM8509^T^

Diphosphatidylglycerol (DPG), phosphatidylcholine (PC), phosphatidylinositol (PI), phosphatidylglycerol (PG), phosphatidylethanolamine (PE), sphingoglycolipid (SGL), unidentified phospholipids (PL), unidentified glycolipid (GL), unidentified aminolipid (AL) and unidentified polar lipid (L)

+, positive; (w), weakly positive; –, negative; ND, no data

*Data from Lee et al. (2017b)

^**^Data from Yoon et al. (2022)

^†^Data from He et al. (2019)

^↓^Data from Nedashkovskaya et al. (2009)

^‡^Data from Park et al. (2020)

### 16S rRNA gene analysis

**Fig. 1:**
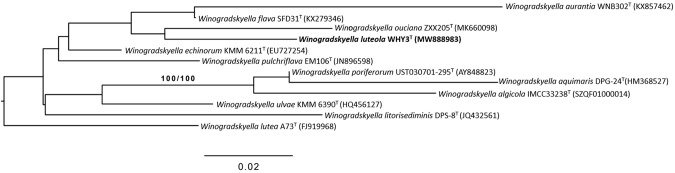
ML tree of strain WHY3^T^ and its closely related type strains inferred under the GTR + GAMMA model and rooted by midpoint-rooting. The numbers above the branches are support values (above 60%) from ML (*left*) and MP (*right*) bootstrapping. The ML and MP bootstrapping average support were 26.12% and 42.40%, respectively

**Fig. 2:**
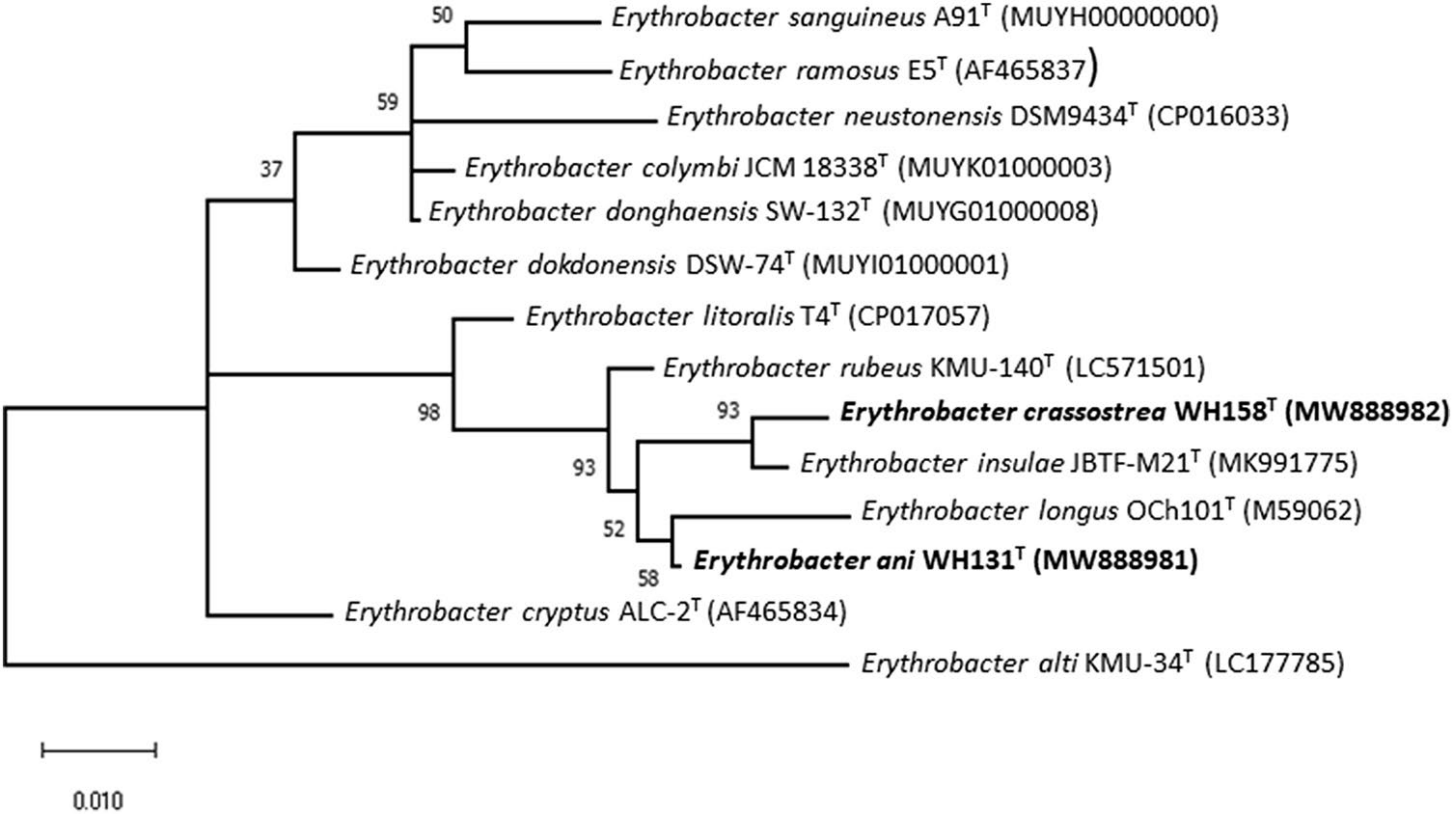
Maximum Likelihood method tree for strains WH131^T^ and WH158^T^, based on 16S rRNA gene sequences. Bootstrap values are expressed as percentages of 100 replications. Bar, 0.010 substitutions per nucleotide position. The tree is drawn to scale, with branch lengths measured in the number of substitutions per site. This analysis involved 14 nucleotide sequences. There were a total of 1493 positions in the final data

According to the EZBioCloud server's results, strains WHY3^T^, WH131^T^, and WH158^T^ were most closely related to the following strain types: 97.6% to *Winogradskyella flava* SFD31^T^, 96.7% to *Winogradskyella echinorum* KMM 6211^T^ for strain WHY3^T^; 99.1% *Erythrobacter longus* OCh101^T^, 98.5% *Erythrobacter insulae* JBTF-M21^T^ for strain WH131^T^; 99.1% *Erythrobacter insulae* JBTF-M21^T^, 98.6% *Erythrobacter longus* OCh101^T^ for strain WH158^T^. 16S rRNA gene results of strain WHY3^T^ from phylogenetic dendrogram demonstrated proximity of 96.5% to *Winogradskyella ouciana* ZXX205^T^. Phylogenetic trees based on 16S rRNA gene sequences of strains WHY3^T^, WH131^T^, and WH158^T^ and its closely related type strains are shown in Fig. 1 and Fig. 2. It shows that strain WHY3^T^ formed a highly supported cluster with *Winogradskyella* species (*W. ouciana* ZXX205^T^ and *W. flava* SFD31^T^). Furthermore, strain WHY3^T^ formed a well-supported branch alongside *W. ouciana* ZXX205^T^; and also strains WH131^T^ and WH158^T^ formed a highly supported cluster with *Erythrobacter* species (*E. rubeus* KMU-140^T^, *E. longus* OCh101^T^and *E. insulae* JBTF-M21^T^); strains WH131^T^ and WH158^T^ formed a branch alongside *E. longus* OCh101^T^ and *E. insulae* JBTF-M21^T^, respectively.

## Chemotaxonomic characterization

The major cellular fatty acids and polar lipids for strain WHY3^T^ were C_15:0_, anteiso-C_15:1_ ω7c, iso-C_15:0_, C_16:1_ω7c, phosphatidylethanolamine, an unknown glycolipid, six unidentified aminolipids, and four unidentified lipids. The major cellular fatty acids and polar lipids for strains WH131^T^ and WH158^T^ were C_14:0_2-OH, t_18:1_ω12, diphosphatidylglycerol, phosphatidylethanolamine, phosphatidylglycerol, two unknown glycolipids, sphingoglycolipids, two phosphatidylcholines, four unidentified lipids, and two unidentified aminolipids for strains WH131^T^ and C_17:0,_ and C_18:1_ω7c, diphosphatidylglycerols, phosphatidylethanolamine, phosphatidylglycerol, unknown glycolipids, sphingoglycolipids, phosphatidylcholine, four unidentified lipids, two unidentified aminolipids for strain WH158^T^ (see Fig. S2 and Table S2). In all three, unknown fatty acids are the dominant constituents. The fatty acid iso-C_15:1_ G is reported as one of the dominant ones in *W. flava* KCTC 52348^T^ and *W. ouciana* ZXX205^T^, but it was absent in strain WHY3^T^ as well as anteiso-C_15:1_ ω7c and C_16:1_ω7c which are present in strain WHY3^T^ but not in *W. flava* KCTC 52348^T^ and *W. ouciana* ZXX205^T^. The fatty acid iso-C_18:0_ is only reported in *Erythrobacter rubeus* KMU-140^T^. C_18:1_ω7c is the largest amount in strain WH158^T^, *E. insulae* JBTF-M21^T^, and *E. rubeus* KMU-140^T^ but was absent in strains WH131^T^, *E. longus* DSM 6997^T^, and *E. litoralis* DSM8509^T^. The fatty acid fatty t_18:1_ω12 represents the largest amount in strain WH131^T^*, E. longus* DSM 6997^T^ and *E. litoralis* DSM8509^T^. C_17:1_ω6c is reported as the major fatty acid in just *E. insulae* JBTF-M21^T^ (see Fig. S2 and Table S2).

Menaquinone-6 (MK-6) was found to be the major respiratory quinone in strain WHY3^T^. Ubiquinone-10 (Q-10) was detected in strains WH131^T^ and WH158^T^.

### Genomic characteristics and phylogenomic analysis

Only one 16S rRNA gene sequence was discovered in the whole-genome data of strains WHY3^T^, WH131^T^, and WH158^T^, showing that the genomic data were not contaminated by other species. The draft assembled genome sequence of strain WHY3^T^ comprised of the following: 3,532,486 bp with a G + C content of 34.4%. The genome included 3276 genes comprising 3194 protein-coding genes, 48 tRNA genes, 3 rRNA genes, and 4 non-coding RNA. In parallel, strains WH131^T^ and WH158^T^ had 3,153,164 bp and 2,586,581 bp with a G + C content of 59.7% and 56.6%, respectively. The genome included 3077 genes comprising 3011 protein-coding genes, 44 tRNA genes, 3 rRNA genes, and 4 non-coding RNA for strain WH131^T^ and 2506 genes comprising 2447 protein-coding genes, 41 tRNA genes, 3 rRNA genes, and 2 non-coding RNA for strain WH158^T^. The phylogenomic tree (Fig. S3) shows that strain WHY3^T^ is well supported by *Winogradskyella* species with pseudo-bootstrap support values > 60% from 100 replications, with average branch support of 65.5%.

The phylogenomic tree (Fig. S4) with average branch support of 40.3% shows that strain WH131^T^ was located in a cluster, although it was not well supported, with *Pseudopontixanthobacter vadosimaris* JL3514^T^ which is not the *Erythrobacter* species. However, the strain WH158^T^ formed a well-defined tight cluster with *Erythrobacter insulae* JBTF-M21^T^, but it was relatively close to *Parerythrobacter jejuensis* JCM16677^T^. Therefore, another investigation was undertaken using whole-proteome-based GBDP distances. As a consequence, a phylogenetic tree with average branch support of 94.5% was produced, which was more trustworthy than the nucleotide-based phylogenomic tree result (Fig. S5) since strains WH131^T^ and WH158^T^ were classified in the same clade as other *Erythrobacter* species with a very high support score. In the whole-proteome-based phylogenetic tree, strains WH131^T^ and WH158^T^ were located in a very high-supported clade together with *E. rubeus* KMU-140^T^ and *E. insulae* JBTF-M21^T^, respectively.

An additional study was conducted using genomic data to identify the genus classification, using the average amino acid identity (AAI) (Rodriguez-R and Konstantinidis, 2014) and the percentage of conserved protein (POCP) values. The POCP values and AAI values between the genomes of strains WH131^T^ and WH158^T^ against *Erythrobacter insulae* JBTF-M21^T^, *Erythrobacter rubeus* KMU-140^T^, *Erythrobacter longus* DSM 6997^T^, *Erythrobacter litoralis* DSM8509^T^, *Pseudopontixanthobacter vadosimaris* JL3514^T^, *Parerythrobacter jejuensis* JCM 16677^T^ are shown in Table 2 with 81.9% (POCP) and 87.6% (AAI) as the highest percentages against *Erythrobacter rubeus* KMU-140^T^ for strain WH131^T^ and 73.0% (POCP) and 81.2% (AAI) as the highest percentages value against *Erythrobacter insulae* JBTF-M21^T^ for strain WH158^T^ (Qin et al. 2014; Rodriguez-R and Konstantinidis 2014). Thus, isolates WH131^T^ and WH158^T^ could be classified as *Erythrobacter* rather than *Pseudopontixanthobacter* or *Parerythrobacter*.

**Table 2:** Genome relatedness between the strains WH131^T^ and WH158^T^, genus *Erythrobacter,* and other closet type strains according to the average amino acid identity (AAI) value and the percentage of conserved protein (POCP) values

Strain	1		2		3		4		5		6	
	POCP %	AAI %	POCP %	AAI %	POCP %	AAI %	POCP %	AAI %	POCP %	AAI %	POCP %	AAI %
*E. ani* WH131^T^ (JAGSPB000000000)	71.5	77.7	**81.9**	**87.6**	65.6	72.3	68.3	73.2	60.2	67.0	65.8	68.3
*E. crassostrea* WH158^T^ (JAGSPC000000000)	**73.0**	**81.2**	64.3	76.7	62.7	71.3	61.3	71.5	54.0	63.7	64.1	66.9
*E. insulae* JBTF-M21^T^ (GCA_007004095)	100	100	**71.3**	**77.6**	69.4	71.8	69.2	72.7	59.5	64.8	67.3	67.8
*E. rubeus* KMU-140^T^ (GCA_014705715)	**71.3**	**77.6**	100	100	64.3	72.0	68.6	73.4	57.2	64.9	66.5	68.4
*P. vadosimaris* JL3514^T^ (GCA_012979275)	59.5	64.8	57.2	64.9	56.0	64.3	58.1	65.4	100	100	61.0	67.6
*P. jejuensis* JCM 16677^T^ (GCA_009827995)	67.3	67.8	66.5	68.4	62.5	66.1	64.5	67.7	61.0	67.6	100	100

1, *Erythrobacter insulae* JBTF-M21^T^; 2, *Erythrobacter rubeus* KMU-140^T^; 3, *Erythrobacter longus* DSM 6997^T^; 4, *Erythrobacter litoralis* DSM8509^T^; 5, *Pseudopontixanthobacter vadosimaris* JL3514^T^; 6, *Parerythrobacter jejuensis* JCM 16677^T^

POCP (percentage of conserved proteins)

AAI (average amino acid identity)

Additionally, as shown in Table S3 and Table S4, all of the type strains had an ANI value less than the species cut-off value of 95% and dDDH scores less than the threshold value of 70%, indicating that strains WHY3^T^, WH131^T^, and WH158^T^ can be distinguished from the other known available *Winogradskyella* and *Erythrobacter* species (Chun et al. 2018).

The genes related to bioremediation using KBase database for *Winogradskyella luteol* WHY3^T^*, Erythrobacter ani* WH131^T^, *Erythrobacter crassostrea* WH158^T^, and closest type strains are reported in Table 3.

**Table 3:** Distribution of genes involved in bioremediation in 1, *Winogradskyella luteola* WHY3^T^; 2, *Winogradskyella flava* KCTC 52348^T^ 3, *Winogradskyella ouciana* ZXX205^T^; 4, *Winogradskyella echinorum* KCTC 22026^T^; 5, *Erythrobacter ani* WH131^T^; 6, *Erythrobacter crassostrea* WH158^T^; 7, *Erythrobacter insulae* JBTF-M21^T^; 8, *Erythrobacter rubeus* KMU-140^T^; 9, *Erythrobacter longus* DSM 6997^T^; 10, *Erythrobacter litoralis* DSM8509^T^ using https://narrative.kbase.us/ accessed on 04 April 2022

Genus name Species name	*Winogradskyella*					*Erythrobacter*					
	1	2	3	4	5		6	7	8	9	10
Target substance and genes											
Inorganic mercury (*mer*A, *mer*B, *Mer*C, *Mer*E, *Mer*F, *Mer*T, *Mer*P)	*mer*T *mer*P	–	*mer*T *mer*P	–	–		–	–	–	–	–
PAH (*phn*A)	*phn*A	*phn*A	*phn*A	*phn*A	–		–	–	–	–	–
Polyhydroxyalkanoic acid	–	–	–	–	+		+	+	+	+	+
Phenol degradation (*dmp*N)	–	–	–	–	–		–	–	–	–	*–*
Chromate resistance (*chr*B)	–	–	–	–	–		–	–	–	–	–
Cobalt–zinc–cadmium resistance protein (*czc*A*, czc*D)	*czc*D	*czc*D *czc*A	*czc*D	*czc*D	*czc*D		*czc*D	*czc*D	*czc*D	*czc*D	*czc*D
Nickel–cobalt–cadmium resistance protein (*ncc*A, *ncc*X)	–	–	–	–	*ncc*X		–	–	–	*ncc*X	–
Copper resistance (*cus*A, *cus*B, *cus*C, *cus*F, protein B)	–	*cus*A	–	–	*cus*A *cus*B *cus*C protein B		–	protein B	protein B	*cus*A *cus*B	protein B

+, presence; –, absence of gene

Mercury resistance in the environment might be due to the presence of *mer*T and *mer*P genes in *Winogradskyella luteol* WHY3^T^ and *Winogradskyella ouciana* ZXX205^T^, as described as a different approach to mercury resistance and bioaccumulation by marine bacteria (Zhang et al. 2020). The presence of *phn*A gene in strain *Winogradskyella luteol* WHY3^T^ and other nearest type strains emphasized the role of *Winogradskyella* for oxidation of anthracene and phenanthrene, although these genes were not detected in *Erythrobacter ani* WH131^T^and *Erythrobacter crassostrea* WH158^T^. The heavy metal resistance protein cobalt–zinc–cadmium *czc*D gene was found in *Winogradskyella luteol* WHY3^T^, *Erythrobacter ani* WH131^T^and *Erythrobacter crassostrea* WH158^T^. A related gene for granulate polyhydroxyalkanoates (PHAs) was present in *Erythrobacter ani* WH131^T^and *Erythrobacter crassostrea* WH158^T^. The genes *cus*A, *cus*B*, cus*C, and protein B (related to Copper resistance genes) and the Nickel–cobalt–cadmium resistance protein genes *ncc*X were reported just for *Erythrobacter ani* WH131^T^, but not for *Erythrobacter crassostrea* WH158^T^ (Table 3).

Additionally, gene annotation using RAST analysis (https://rast.nmpdr.org) predicted 3319, 3147, and 2564 coding sequences in the genome of strains WHY3^T^, WH131^T^and WH158^T^, respectively. The dominant fraction of subsystem features for strain WHY3^T^ were amino acids and derivatives (169), Cofactors–Vitamins–Prosthetic Groups–Pigments (123), protein metabolism (139), carbohydrates (93), Fatty Acids–Lipids, and Isoprenoids (50). Other genes which were detected that have a role in the development process, were present as follows: virulence, disease, and defense (24), stress Response (20), and metabolism of Aromatic Compounds (9). For the protein metabolism genes, a significant percentage was for Protein biosynthesis. The dominant fraction of subsystem features for strains WH131^T^ and WH158^T^ were amino acids and derivatives (214,158), protein metabolism (164, 84), carbohydrates (136,91), Cofactors–Vitamins–Prosthetic Groups–Pigments (110,94), Membrane Transport (100,42), Fatty Acids–Lipids, and Isoprenoids (80,65), Respiration (83,63) and Stress Response (51,31), respectively. The presence of protein and nucleoprotein secretion system gene (Type IV) was remarkable in strain WH131^T^ as it is absent in strain WH158^T^ (Fig. S6) (Table S5). The extract of strain WHY3^T^ could moderately inhibit the growth of *Staphylococcus aureus* Newman, *Candida albicans* DSM 1665 and weak inhibited *Bacillus subtilis* DSM 10 and *Wickerhamomyces anomalus* DSM 6766; the extract of strains WH131^T^ and WH158^T^ showed no remarkable inhibition against the most tested microbes (Table 6).

## Conclusion

This polyphasic study indicates that isolates WHY3^T^, WH131^T^, and WH158^T^ are new species belonging to the genus *Winogradskyella* and *Erythrobacter*. Based on our results, we propose the name *Winogradskyella luteol* sp.nov. for strain WHY3^T^; and *Erythrobacter ani* sp.nov. and *Erythrobacter crassostrea* sp.nov. for strains WH131^T^ and WH158^T^, respectively. Environmental pollution is one of the most serious issues that the twenty-first century is dealing with. Restoration and rehabilitation of contaminated sites have attracted a great deal of interest from the scientific community, with bioremediation as the main methods in such endeavors. Based on our genome analysis, all three strains, WHY3^T^, WH131^T^, and WH158^T^, show they have the potential for bioremediation, as they contain certain important genes that have already been proven to be involved in bioremediation processes.

## Description of *Winogradskyella luteola *sp.nov.

*Winogradskyella luteola* (lu.te.o'la. L. fem. adj. luteola, light yellow).

Cells are Gram-negative-staining, motile by gliding, rod-shaped, aerobic, no-spore-form, devoid of flagella, 0.3–0.4 µm width and 0.8–2.1 µm in length. Colonies are yellow. Temperature range for growth is 5–40 (℃) pH spectrum for growth 6–9 and NaCl (optimum) for growth 2.5(%).

Positive for: catalase, oxidase, esterase (C4), esterase lipase (C8), phosphatase acid, naphtol-AS-BI-phosphohydrolase, acetoin, phosphatase alkaline, leucin arylamidase, valine arylamidase, assimilation of d-glucose, malic acid. Positive results (Biolog GEN III Micro Plate analysis) for glucuronamide, Gentiobiose, d-turanose, α-d-glucose, d-fucose, l-fucose, l-rhamnose, acetoacetic acid, acetic acid, l-malic acid, bromo-succinic acid, potassiumtellurite, sodium butyrate, sodium bromate, d-glucuronic acid, d-fructose-6-PO4, l-histidine, 1% NaCl, 4% NaCl and 8% NaCl, 1% sodium lactate, fusidic acid, d-serine, troleandomycin, rifamycin SV, minocycline, lincomycin, guanidine HCl, niaproof 4, vancomycin, nalidixic as well as susceptibility for chloramphenicol, thiostrepton, erythromycin, aztreonam. Major polar lipids are phosphatidylethanolamine (PE), unidentified glycolipid (GL), unidentified aminolipid (AL), and unidentified polar lipid (L). The predominant cellular fatty acids are C15:0, anteiso-C15:1 ω7c, iso-C15:0, C16:1ω7c. The menaquinone-6 (MK-6) is the major respiratory quinone. The DNA G + C content of type strain is 34.4%. Genome size of strain WHY3^T^ indicates 3,53 Mbp.

The type strain WHY3^T^ (= DSM 111804^ T^ = NCCB 100833^ T^) was isolated from Hemolymph of Pacific Oyster *Crassostrea gigas,* which was collected from Wilhelmshaven in Germany.

The GenBank/NCBI accession numbers for 16S rRNA Gene sequence and whole-genome sequence of strain WHY3^T^ are MW888983 and JAGSPD000000000, respectively.

## Description of *Erythrobacter ani* sp.nov.

*Erythrobacter ani* (a'ni. L. gen. n. *ani*, of the anus, referring to anus area near the adductor muscle in *Crassostrea gigas*).

Gram-negative, no-spore-form, non-flagellated and coccoid, ovoid or rod-shaped cell, 0.2–0.3 µm width and 0.6–3.1 µm length: Colonies are yellowish-orange. Temperature range for growth is 20–35 (℃) pH spectrum for growth 6–9 and NaCl (optimum) for growth 2.5(%). Positive for: catalase, oxidase, esterase (C4), esterase lipase (C8), cystine arylamidase, trypsin, α –chymotrypsin, phosphatase acid, nitrate reduction, arginine dihydrolase, aesculin hedrolysis, tween 80 hydrolysis, phosphatase alkaline, leucin arylamidase, valine arylamidase, assimilation of d-glucose, d-mannose, d-mannitol, d-maltose, malic acid, phenylacetic acid. There are positive results (Biolog GEN III Micro Plate analysis) for glucuronamide, d-glucose-6-phosphate, d-fructose-6-phosphate, d-galacturonic acid, l-lactic acid, α-ketoglutaric acid, β-hydroxy-D, l-butyric acid, acetic acid, dextrin, d-maltose, d-trehalose, d-cellobiose, gentiobiose, sucrose, d-turanose, stachyose, d-raffinose, α- d-lactose, d-melibiose, β-methyl-d-glucoside, d-salicin, 1% NaCl, α-d-glucose, d-mannose, d-fructose, d-galactose, d-fucose, l-fucose, l-rhamnose, inosine, d-sorbitol, d-mannitol, d-aspartic acid, gelatin, glycyl-l-proline, l-glutamic acid, pectin, l-galactonic acid lactone, d-gluconic acid, d-glucuronic acid, mucic acid, quinic acid, methyl pyruvate, d-lactic acid methyl ester, d-malic acid, l-malic acid, bromosuccinic acid, p-Hydroxy phenylacetic acid, tween 40, α-ketobutyric acid, formic acid, potassium tellurite, aztreonam, sodium butyrate. The other substrates were inactive. Antibiotic susceptibility for gentamycin, kanamycin, chloramphenicol, spectinomycin, fusidic acid, thiostrepton, erythromycin and tetracycline. Major polar lipids are diphosphatidylglycerol (DPG), phosphatidylethanolamine (PE), phosphatidylcholine (PC), phosphatidylglycerol (PG), sphingoglycolipid (SGL), unidentified glycolipid (GL), unidentified aminolipid (AL), and unidentified polar lipid (L). The predominant cellular fatty acids are C_14:0_2-OH, t_18:1_ω12. The ubiquinone-10 (Q-10) is the predominant isoprenoid quinone. The DNA G + C content of type strain is 59.7%. Genome size of strain WHY3^T^ indicates 3,15 Mbp.

The type strain WH131^T^ (= DSM 112099^T^ = NCCB 100824^T^) was isolated from Hemolymph of Pacific Oyster *Crassostrea gigas,* which was collected from Wilhelmshaven in Germany.

The GenBank/NCBI accession numbers for 16S rRNA Gene sequence and whole-genome sequence of strain WH131^T^ are MW888981 and JAGSPB000000000, respectively.

## Description of *Erythrobacter crassostreae* sp.nov.

*Erythrobacter crassostreae* (crass.os'tre.ae. N.L. gen. n. *crassostreae*, referring to the giant oyster *Crassostrea gigas*).

Gram-negative, no-spore-form, non-flagellated, and coccoid, ovoid or rod-shaped cell, 0.2–0.4 µm width and 0.5–2.4 µm length. Colonies are orange. Temperature range for growth is 20–35 (℃) pH spectrum for growth 6–9 and NaCl (optimum) for growth 2.5(%). Positive for: catalase, oxidase, esterase (C4), esterase lipase (C8), cystine arylamidase, trypsin, α-chymotrypsin, phosphatase acid, lipase (C14), Naphtol-AS-BI-phosphohydrolase, arginine dihydrolase, aesculin hedrolysis, phosphatase alkaline, leucin arylamidase, valine arylamidase, phosphatase alkaline, leucin arylamidase, valine arylamidase, assimilation of d-glucose, d-mannose, d-mannitol, d-maltose, phenylacetic acid. There are positive results (Biolog GEN III Micro Plate analysis) for dextrin, d-maltose, d-trehalose, d-cellobiose, gentiobiose, sucrose, d-turanose, stachyose, d-raffinose, α- d-lactose, d-melibiose, 1% NaCl, α-d-glucose, d-mannose, d-fructose, d-galactose, l-fucose, d-fucose, l-rhamnose, inosine, d-mannitol, d-glucose-6-phosphate, d-fructose-6-phosphate, glycyl-l-proline, l-glutamic acid, pectin, d-galacturonic acid, l-galactonic acid lactone, d-glucuronic acid, d-gluconic acid, glucuronamide, mucic acid, quinic acid, *p*-Hydroxy phenylacetic acid, methyl pyruvate, l-lactic acid, d-malic acid, l-malic acid, nalidixic acid, tween 40, α-ketobutyric acid, acetic acid, formic acid, aztreonam, sodium butyrate. The other substrates were inactive. Antibiotic susceptibility for gentamycin, kanamycin, chloramphenicol, spectinomycin, fusidic acid, thiostrepton, nalidixic acid, erythromycin, and tetracycline. Major polar lipids are diphosphatidylglycerol (DPG), phosphatidylethanolamine (PE), phosphatidylcholine (PC), phosphatidylglycerol (PG), sphingoglycolipid (SGL), unidentified glycolipid (GL), unidentified aminolipid (AL), and unidentified polar lipid (L). The predominant cellular fatty acids are C_17:0_ and C_18:1_ω7c. The ubiquinone-10 (Q-10) is their predominant isoprenoid quinone. The DNA G + C content of type strain is 56.6%. The genome size of strain WHY3^T^ indicates 2,58 Mbp.

The type strain WH158^T^ (= DSM 112102^ T^ = NCCB 100877^ T^) was isolated from Hemolymph of Pacific Oyster *Crassostrea gigas,* which was collected from Wilhelmshaven in Germany.

The GenBank/NCBI accession numbers for 16S rRNA Gene sequence and whole-genome sequence of strain WH158^T^ are MW888982 and JAGSPC000000000, respectively.

## Supplementary Information

Below is the link to the electronic supplementary material.

Supplementary file1 (DOCX 1894 KB)

## Data Availability

Not applicable.
