# Supplementary material for: Winogradskyella luteola sp.nov., Erythrobacter ani sp. nov., and Erythrobacter crassostrea sp.nov., isolated from the hemolymph of the Pacific Oyster Crassostrea gigas
Source: Arch Microbiol. 2022 Jul 14;204(8):488. doi: 10.1007/s00203-022-03099-y (PMC9283347; doi:10.1007/s00203-022-03099-y)
Supplement: Supplementary file 1 — Supplementary file1 (DOCX 1894 KB) [file 203_2022_3099_MOESM1_ESM.docx]

**Supplementary materials**

***Winogradskyella luteola*sp.nov., *Erythrobacter ani* sp. nov., and *Erythrobacter crassostrea* sp.nov., isolated from the hemolymph of the Pacific Oyster *Crassostrea gigas***

**Hani Pira^a*^, Chandra Risdian^a,e^, Mathias Müsken ^b^, Peter J. Schupp ^c,d^ and Joachim Wink ^a^***

^a^ Microbial Strain Collection (MISG), Helmholtz Centre for Infection Research (HZI), 38124 Braunschweig, Germany; [hani.pira@helmholtz-hzi.de (H.P)](mailto:hani.pira.com%20(H.P)); [Chandra.Risdian@helmholtz-hzi.de](mailto:Chandra.Risdian@helmholtz-hzi.de) (C.R); [Joachim.Wink@helmholtz-hzi.de](mailto:Joachim.Wink@helmholtz-hzi.de) (J.W).

^b^ Central Facility for Microscopy, Helmholtz Centre for Infection Research (HZI), 38124 Braunschweig, Germany; [Mathias.Muesken@helmholtz-hzi.de](mailto:Mathias.Muesken@helmholtz-hzi.de)(M.M).

^c^ Environmental Biochemistry, Institute for Chemistry and Biology of the Marine Environment (ICBM), Oldenburg, Germany; [peter.schupp@uni-oldenburg.de](file:///\\neon\hpi19$\article%20HANI\Diversity%20format\peter.schupp@uni-oldenburg.de) (PJ.S).

^d^ Helmholtz Institute for Functional Marine Biodiversity at the University of Oldenburg, Ammerländer Heerstraße 231, 26129 Oldenburg, Germany (PJ.S).

^e^ Research Center for Environmental and Clean Technology, National Research and Innovation Agency (BRIN), Bandung 40135, Indonesia


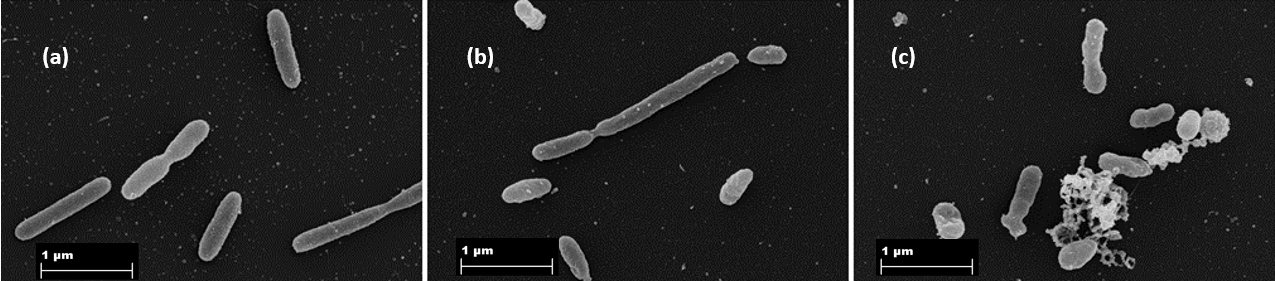


**Fig. S1**. Scanning electron microscopy image of strains (a) WHY3^T^, (b) WH131^T^, and (c) WH158^T^.


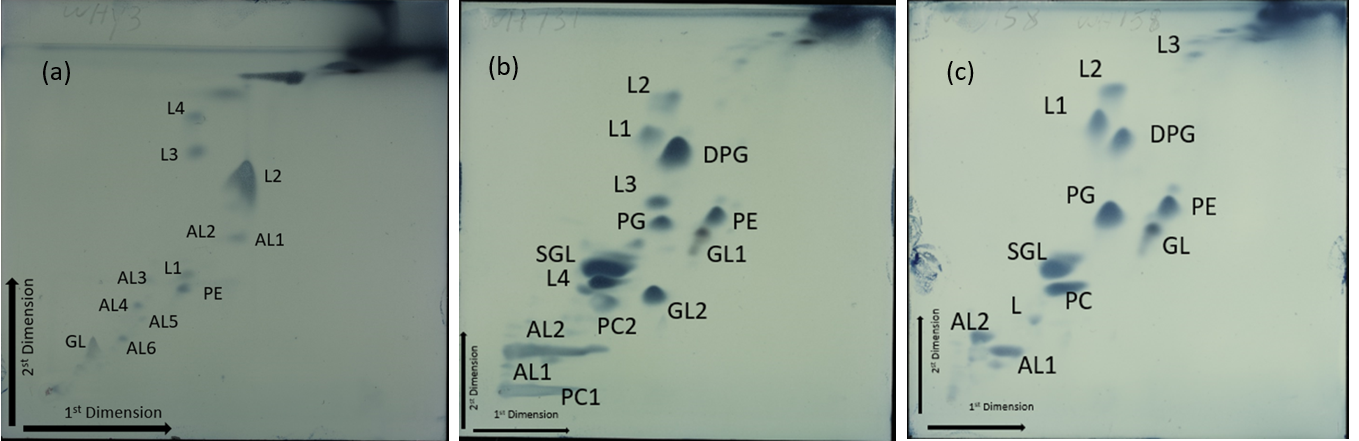


**Fig. S2**.The profile of cellular polarlipid of strains (a) WHY3^T^, (b) WH131^T^, (c) WH158^T^. Diphosphatidylglycerol (DPG), phosphatidylcholine (PC), phosphatidylinositol (PI), phosphatidylglycerol (PG), phosphatidylethanolamine (PE), sphingoglycolipid (SGL), unidentified phospholipids (PL), unidentified glycolipid (GL), unidentified aminolipid (AL) and unidentified polar lipid (L).

**Table S1**

Antibiotic susceptibility of strains WHA3^T^, WH131^T,^ and WH158^T^ for the most closely related type strains. Strains: 1, *Winogradskyella luteola* WHY3^T^; 2, *Winogradskyella flava* KCTC 52348^T^ 3, *Winogradskyella ouciana* ZXX205^T^; 4, *Winogradskyella echinorum* KCTC 22026^T^; 5, *Erythrobacter ani* WH131^T^; 6, *Erythrobacter crassostrea* WH158^T^; 7, *Erythrobacter insulae* JBTF-M21^T^; 8, *Erythrobacter rubeus* KMU-140^T^; 9, *Erythrobacter longus* DSM 6997^T^; 10, *Erythrobacter litoralis* DSM8509^T^.

| **Genus name**  **Species name** | ***Winogradskyella*** | | | | ***Erythrobacter*** | | | | | |
| --- | --- | --- | --- | --- | --- | --- | --- | --- | --- | --- |
|  | **1** | **2** | **3** | **4** | **5** | **6** | **7** | **8** | **9** | **10** |
| Susceptibility to: |  |  |  |  |  |  |  |  |  |  |
| Polymyxin | - | ND | ND | -⸸ | - | - | -‡ | ND | - | - |
| Gentamycin | - | -* | ND | -⸸ | + | + | -‡ | ND | + | - |
| Oxytetracycline | - | ND | ND | ND | - | - | ND | ND | - | - |
| Ampicillin | - | +* | ND | -⸸ | - | - | -‡ | ND | - | - |
| Chloramphenicol | + | +* | ND | +⸸ | + | + | +‡ | ND | + | + |
| Spectinomycin | - | ND | ND | -⸸ | + | + | ND | ND | + | + |
| Kanamycin | - | -* | ND | -⸸ | + | + | -‡ | ND | + | + |
| Cephalosporin | - | ND | ND | ND | - | - | ND | ND | - | - |
| Fusidic acid | + | ND | +† | ND | + | + | ND | -** | + | + |
| Bacitracin | - | ND | ND | ND | - | - | ND | ND | - | - |
| Thiostrepton | + | ND | ND | ND | + | + | ND | ND | + | + |
| Trimethoprim | - | ND | ND | ND | - | - | ND | ND | - | - |
| Erythromycin | + | +* | ND | +⸸ | + | + | ND | ND | + | + |
| Tetracycline | - | ND | ND | -⸸ | + | + | +‡ | ND | + | + |
| Vancomycin | + | +* | -† | ND | - | - | ND | -** | - | - |
| Amikacin | ND | -* | ND | ND | ND | ND | ND | ND | ND | ND |
| Nalidixic acid | + | -* | +† | ND | - | + | ND | -** | - | + |
| Penicillin | ND | -* | ND | ND | ND | ND | -‡ | ND | + | - |
| Streptomycin | - | -* | ND | ND | ND | ND | -‡ | ND | - | - |
| Lincomycin | + | ND | - | +⸸ | - | - | -‡ | -** | - | - |
| Troleandomycin | + | ND | +† | ND | - | - | ND | -** | - | - |
| Rifamycin SV | + | ND | +† | ND | - | - | ND | -** | - | - |
| Minocycline | + | ND | +† | ND | - | - | ND | -** | - | - |
| Aztreonam | + | ND | +† | ND | + | + | ND | -** | - | + |

+, positive; -, negative; ND, no data

**Table S2.** The fatty acid content of strains WHY3^T^, WH131^T^, and WH158^T^ and their most closely related type strains. Strains: 1, *Winogradskyella luteola* WHY3^T^; 2, *Winogradskyella flava* KCTC 52348^T^ 3, *Winogradskyella ouciana* ZXX205^T^; 4, *Erythrobacter ani* WH131^T^; 5, *Erythrobacter crassostrea* WH158^T^; 6, *Erythrobacter insulae* JBTF-M21^T^; 7, *Erythrobacter rubeus* KMU-140^T^; 8, *Erythrobacter longus* DSM 6997^T^; 9, *Erythrobacter litoralis* DSM8509^T^.

| **Genus name**  **Species name** | ***Winogradskyella*** | | | | ***Erythrobacter*** | | | | | |
| --- | --- | --- | --- | --- | --- | --- | --- | --- | --- | --- |
|  | **1** | **2*** | **3**† | **4** | | **5** | **6**‡ | **7**** | **8** | **9** |
| Fatty acid |  |  |  |  | |  |  |  |  |  |
| C_14:0_ | -- | -- | -- | -- | | -- | -- | -- | -- | 5.3 |
| C_15:0_ | **11.5** | 4.2 | -- | -- | | -- | -- | -- | -- | -- |
| C_16:0_ | 4.4 | TR | 3.8 | 2.5 | | 3.2 | 6.6 | **12.5** | 4.1 | **12.5** |
| C_17:0_ | -- | -- | -- | 2.3 | | **8.8** | -- | 3.9 | 2.1 | RT |
| C_18:0_ | 2.9 | -- | 1.2 | 1.6 | | -- | -- | -- | RT |  |
| C_19:0_ | -- | -- | -- | TR | | -- | -- | -- | -- | -- |
| anteiso-C_15:0_ | 1.3 | 2.0 | 4.7 | -- | | -- | -- | -- | 4.2 | -- |
| anteiso- C_15:1_ A | -- | 1.4 | 1.0 | -- | | -- | -- | -- | -- | -- |
| anteiso-C_17:0_ | -- | -- | -- | 2.3 | | 2.6 | -- | -- | **10.0** | 1.2 |
| anteiso-C_15:1_ ω7c | **17.7** | -- | -- | -- | | -- | -- | -- | -- | -- |
| iso-C_14:0_ | -- | 2.6 | 3.9 | -- | | -- | -- | -- | -- | -- |
| iso-C_15:0_ | **17.0** | **20.0** | **23.4** | -- | | -- | -- | -- | -- | -- |
| iso- C_15:0_ 3-OH | -- | 5.5 | 3.6 | -- | | -- | -- | -- | -- | -- |
| iso- C_15:1_ G | -- | **26.6** | **13.7** | -- | | -- | -- | -- | -- | -- |
| iso-C_16:0_ | 1.7 | 1.7 | **10.6** | -- | | -- | -- | -- | -- | -- |
| iso- C_16:0_ 3-OH | -- | 2.6 | **10.7** | -- | | -- | -- | -- | -- | -- |
| iso- C_16:1_ G | -- | -- | 1.6 | -- | | -- | -- | -- | -- | -- |
| iso-C_16:1_ω6c | -- | -- | -- | TR | | -- | -- | -- | -- | -- |
| iso-C_17:0_ | -- | -- | -- | -- | | -- | 4.0 | -- | RT |  |
| iso- C_17:0_ 3-OH | -- | 4.2 | 8.8 | -- | | -- | -- | -- | -- | -- |
| iso-t_17:1_ω7 | 2.8 | -- | -- | -- | | -- | -- | -- | -- | -- |
| iso-C_18:0_ | -- | -- | -- | -- | | -- | -- | **13.4** | -- | -- |
| iso-C_18:1_ω6c | 1.3 | -- | -- | -- | | -- | -- | -- | -- | -- |
| C_16:1_ω7c | **13.2** | -- | -- | 3.4 | | -- | -- | -- | RT | **7.2** |
| C_17:1_ω6c | -- | -- | 1.2 | TR | | 2.3 | **23.9** | 2.0 | -- | 1.9 |
| C_17:1_ω8c | -- | -- | -- | -- | | 3.4 | 2.7 | 3.4 | 2.4 | -- |
| C_18:1_ω5c | -- | -- | -- | -- | | -- | TR | -- | -- | -- |
| C_18:1_ω7c | -- | -- | -- | -- | | **40.0** | **44.0** | **40.9** | -- | -- |
| C_19:1_ω8c | -- | -- | -- | -- | | -- | -- | -- | 1.9 | -- |
| C_12:0_3-OH | -- | -- | 1.2 | -- | | -- | -- | -- | -- | -- |
| C_14:0_2-OH | -- | TR | -- | **8.1** | | 2.4 | 6.1 | 6.6 | **9.6** | 4.7 |
| C_15:0_2-OH | -- | -- | TR | -- | | -- | 5.1 | 5.8 |  |  |
| C_16:0_2-OH | -- | -- | -- | -- | | -- | TR | -- | 1.1 | RT |
| t_18:1_ω12 | -- | -- | -- | **52.6** | | -- | -- | -- | **53.79** | **65.93** |
| t_16:1_ω7 | -- | -- | -- |  | | -- | -- | -- | -- | RT |
| cyclo-C_19:0_d8,9 | -- | -- | -- | TR | | -- | -- | -- | -- | -- |
| 11-methyl C_18:1_ ω7c | -- | -- | -- | -- | | -- | 5.9 | 6.9 | -- | -- |
| Unknown fatty acid | **26.4** | **17.1** | -- | **24.0** | | **37.7** | -- | -- | **17.4** | 4.3 |

TR, trace amount (<1%);--: Not detecte or Not reported

* Data from Lee et al. (Lee et al. 2017b)

† Data from He et al. (He et al. 2019)

‡ Data from [Park](https://www.microbiologyresearch.org/search?value1=Olga+I.+Nedashkovskaya&option1=author&noRedirect=true) et al. (Park et al. 2020)

** Data from Yoon et al. (Yoon et al. 2022)


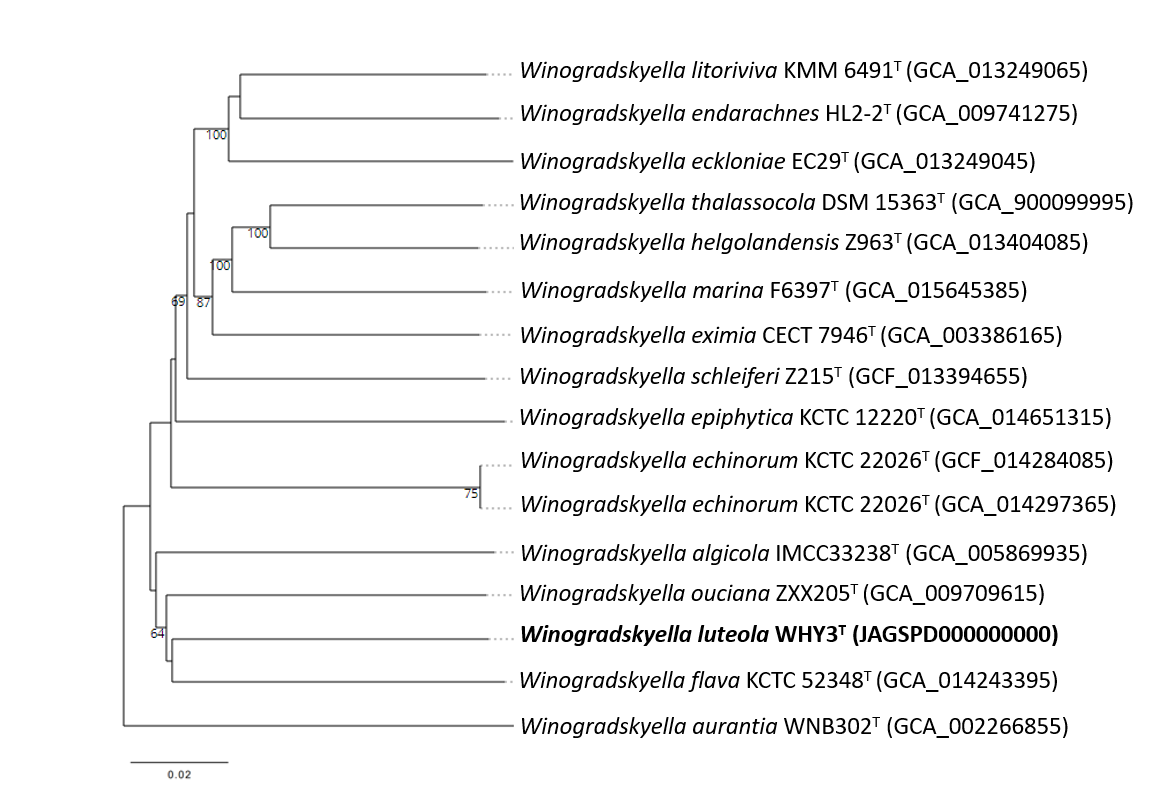


**Fig. S3.** Phylogenomic tree showing relationships between strain WHY3^T^ and the most closely related type strains from the genus *Winogradskyella*. The number at the nodes are GBDP pseudo-bootstrap support values > 60% from 100 replications, with average branch support of 65.5%. Bar, 0.02 substitutions per site.


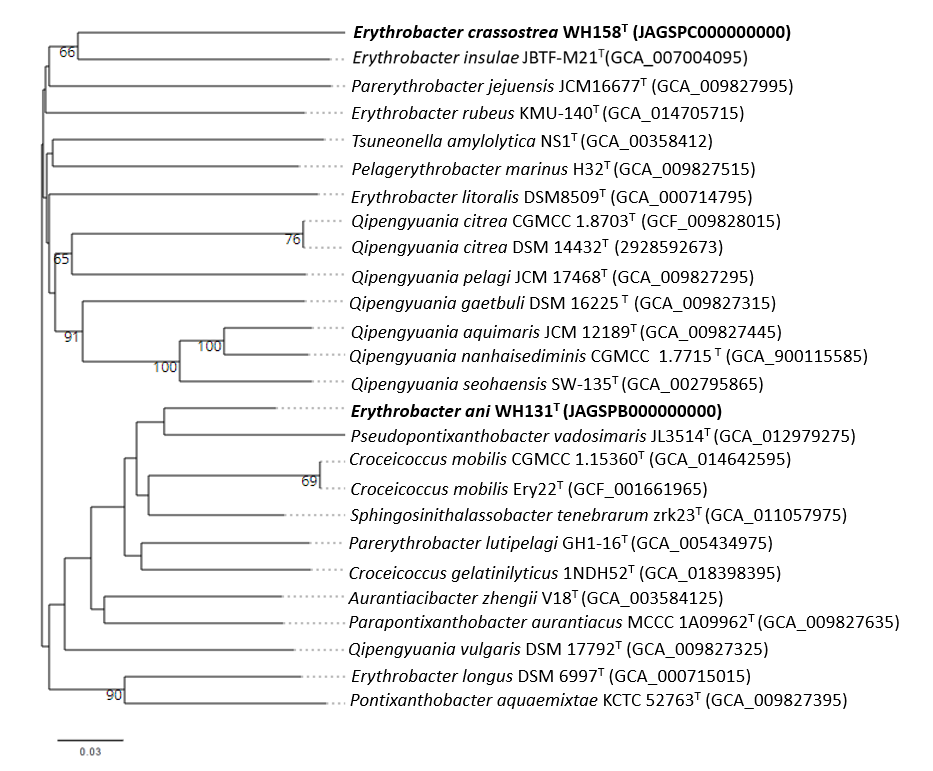


**Fig. S4.** Phylogenomic tree inferred from GBDP distances based on genomic data between strain WH131^T^,WH158^T^ and the closely related type strains based on Type (Strain) Genome Server (TYGS) (https://tygs.dsmz.de/). The number at the nodes are GBDP pseudo-bootstrap support values > 60 % from 100 replications, with average branch support of 40.3%.


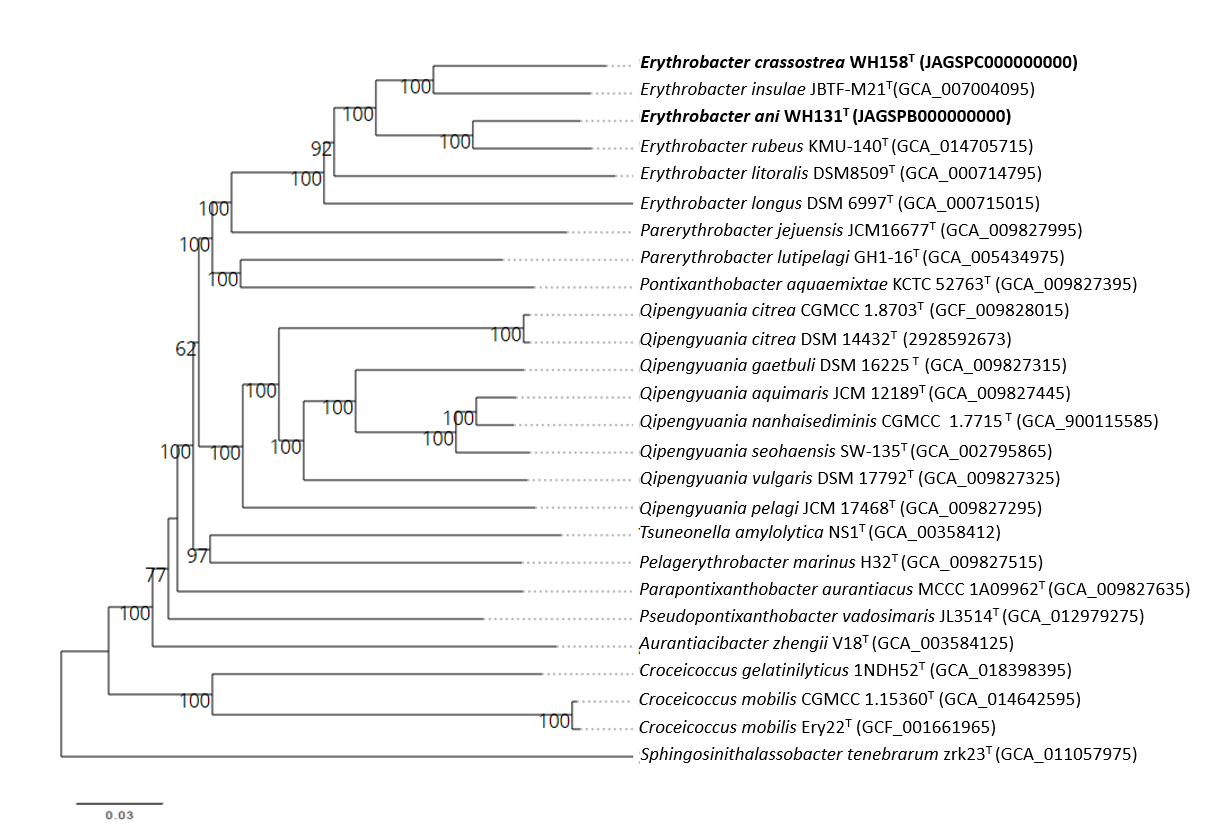


**Fig. S5**. Phylogenomic tree showing relationships between strains WH131^T^, WH158^T^ and the most closely related type strains from the genus *Erythrobacter* according to whole-proteome-base. The number at the nodes are GBDP pseudo-bootstrap support values > 60% from 100 replications, with average branch support of 94.5%.

**Table S3.** ANI values of the strains WHY3^T^, WH131^T^and WH158^T^ and its closely related type strains. 1, *Winogradskyella luteola* WHY3^T^; 2, *Winogradskyella* *flava* KCTC 52348^T^; 3, *Winogradskyella ouciana* ZXX205^T^;4,*Winogradskyella echinorum* KCTC 22026^T^; 5, *Erythrobacter ani* WH131^T^; 6, *Erythrobacter crassostrea* WH158^T^; 7, *Erythrobacter insulae* JBTF-M21^T^; 8, *Erythrobacter rubeus* KMU-140^T^; 9, *Erythrobacter longus* DSM 6997^T^; 10, *Erythrobacter litoralis* DSM8509^T^;11, *Pseudopontixanthobacter vadosimaris* JL3514^T^;12, *Parerythrobacter jejuensis* JCM 16677^T^.

| **Genus name**  **Species name** | ***Winogradskyella*** | | | | | ***Erythrobacter*** | | | | | | | |
| --- | --- | --- | --- | --- | --- | --- | --- | --- | --- | --- | --- | --- | --- |
|  | **1**  **ANI** | **2**  **ANI** | **3**  **ANI** | **4**  **ANI** | **5**  **ANI** | | **6**  **ANI** | **7**  **ANI** | **8**  **ANI** | **9**  **ANI** | **10**  **ANI** | **11**  **ANI** | **12 ANI** |
| ***W. luteola* WHY3^T^ (JAGSPD000000000)** | **100** | 77.5 | 77.9 | 77.4 | -- | | -- | -- | -- | -- | -- | -- | -- |
| *W. flava* KCTC 52348^T^ (GCA_014243395) | 77.53 | **100** | 77.7 | 76.7 | -- | | -- | -- | -- | -- | -- | -- | -- |
| *W. ouciana* ZXX205^T^ (GCA_009709615) | 77.9 | 77.7 | **100** | 77.1 | -- | | -- | -- | -- | -- | -- | -- | -- |
| *W. echinorum* KCTC 22026^T^ (GCA_014297365) | 77.4 | 76.7 | 77.1 | **100** | -- | | -- | -- | -- | -- | -- | -- | -- |
| ***E. ani* WH131^T^**  **(JAGSPB000000000)** | -- | -- | -- | -- | **100** | | 74.9 | 75.0 | 81.7 | 72.9 | 74.6 | 73.6 | 71.7 |
| ***E.*** ***crassostrea* WH158^T^**  **(JAGSPC000000000)** | -- | -- | -- | -- | 74.9 | | **100** | 77.4 | 74.9 | 72.4 | 72.8 | 70.1 | 70.9 |
| *E. insulae* JBTF-M21^T^ (GCA_­007004095) | -- | -- | -- | -- | 75.0 | | 77.4 | **100** | 75.2 | 72.5 | 72.9 | 70.3 | 71.1 |
| *E.rubeus* KMU-140^T^ (GCA_014705715) | -- | -- | -- | -- | 81.7 | | 74.9 | 75.2 | **100** | 73.0 | 75.5 | 71.5 | 72.0 |
| *E. longus* DSM 6997^T^ (GCA_000715015) | -- | -- | -- | -- | 72.9 | | 72.4 | 72.5 | 73.0 | **100** | 72.5 | 70.7 | 70.2 |
| *E. litoralis* DSM8509^T^ (GCA_000714795) | -- | -- | -- | -- | 74.6 | | 72.8 | 72.9 | 75.5 | 72.5 | **100** | 72.4 | 72.1 |

OrthoANIu (ANI) values ≥ 95% demonstrated in bold.

**Table S4.** dDDH values of the strains *Winogradskyella* sp WHY3^T^, *Erythrobacter* sp WH131^T^, *Erythrobacter* sp WH158^T,^ and its closely related type strains. 1, *Winogradskyella luteola* WHY3^T^; 2, *Winogradskyella flava* KCTC 52348^T^; 3*, Winogradskyella ouciana* ZXX205^T^;4, *Winogradskyella echinorum* KCTC 22026^T^; 5*, Erythrobacter ani* WH131^T^; 6, *Erythrobacter crassostrea* WH158^T^; 7, *Erythrobacter insulae* JBTF-M21^T^; 8, *Erythrobacter rubeus* KMU-140^T^; 9, *Erythrobacter longus* DSM 6997^T^; 10, *Erythrobacter litoralis* DSM8509^T^; 11, *Pseudopontixanthobacter vadosimaris* JL3514^T^;12, *Parerythrobacter jejuensis* JCM 16677^T^.

| **Genus name**  **Species name** | ***Winogradskyella*** | | | | | ***Erythrobacter*** | | | | | | | |
| --- | --- | --- | --- | --- | --- | --- | --- | --- | --- | --- | --- | --- | --- |
|  | **1**  **dDDH** | **2**  **dDDH** | **3**  **dDDH** | **4**  **dDDH** | **5**  **dDDH** | | **6**  **dDDH** | **7**  **dDDH** | **8**  **dDDH** | **9**  **dDDH** | **10**  **dDDH** | **11**  **dDDH** | **12 dDDH** |
| ***W. luteola*WHY3^T^ (JAGSPD000000000)** | **100** | 20.9 | 21.0 | 20.5 | -- | | -- | -- | -- | -- | -- | -- | -- |
| *W. flava* KCTC 52348^T^ (GCA_014243395) | 20.9 | **100** | 20.7 | 20.1 | -- | | -- | -- | -- | -- | -- | -- | -- |
| *W. ouciana* ZXX205^T^ (GCA_009709615) | 21.0 | 20.7 | **100** | 20.4 | -- | | -- | -- | -- | -- | -- | -- | -- |
| *W. echinorum* KCTC 22026^T^ (GCA_014297365) | 20.5 | 20.1 | 20.4 | **100** | -- | | -- | -- | -- | -- | -- | -- | -- |
| ***E. ani* WH131^T^**  **(JAGSPB000000000)** | -- | -- | -- | -- | **100** | | 18.3 | 18.5 | 23.5 | 19.2 | 18.9 | 31.8 | 19.0 |
| ***E. crassostrea* WH158^T^**  **(JAGSPC000000000)** | -- | -- | -- | -- | 18.3 | | **100** | 19.8 | 18.7 | 17.5 | 17.9 | 18.8 | 17.9 |
| *E. insulae* JBTF-M21^T^ (GCA_­007004095) | -- | -- | -- | -- | 18.5 | | 19.8 | **100** | 18.5 | 18.8 | 18.0 | 18.7 | 18.6 |
| *E.rubeus* KMU-140^T^ (GCA_014705715) | -- | -- | -- | -- | 23.5 | | 18.7 | 18.5 | **100** | 18.9 | 19.1 | 19.4 | 18.8 |
| *E. longus* DSM 6997^T^ (GCA_000715015) | -- | -- | -- | -- | 19.2 | | 17.5 | 18.8 | 18.9 | **100** | 18.6 | 20.8 | 19.0 |
| *E. litoralis* DSM8509^T^ (GCA_000714795) | -- | -- | -- | -- | 18.9 | | 17.9 | 18.0 | 19.1 | 18.6 | **100** | 18.4 | 18.6 |

dDDH values > 70% demonstrated in bold.

**
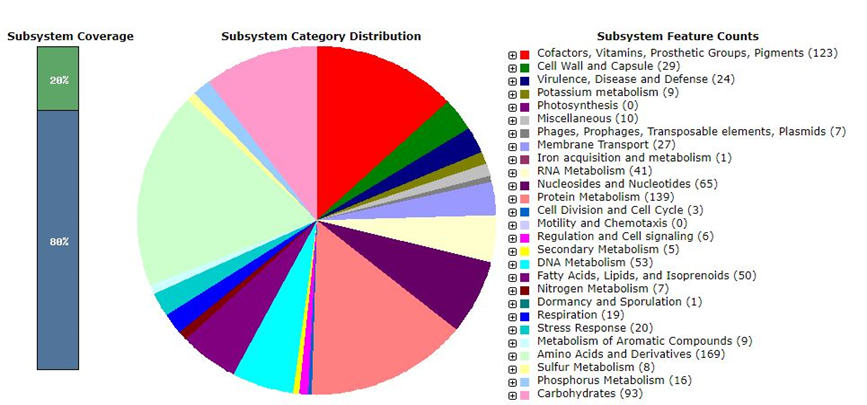
**

(a)

**
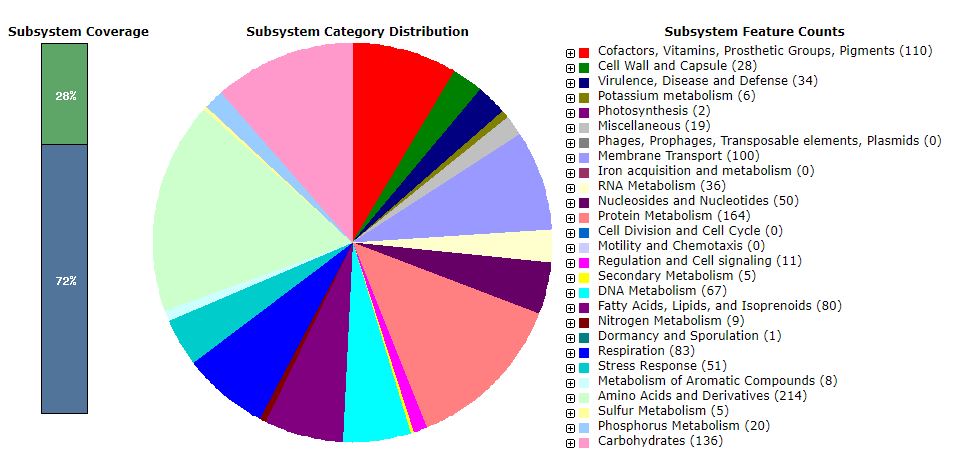
**

(b)

**
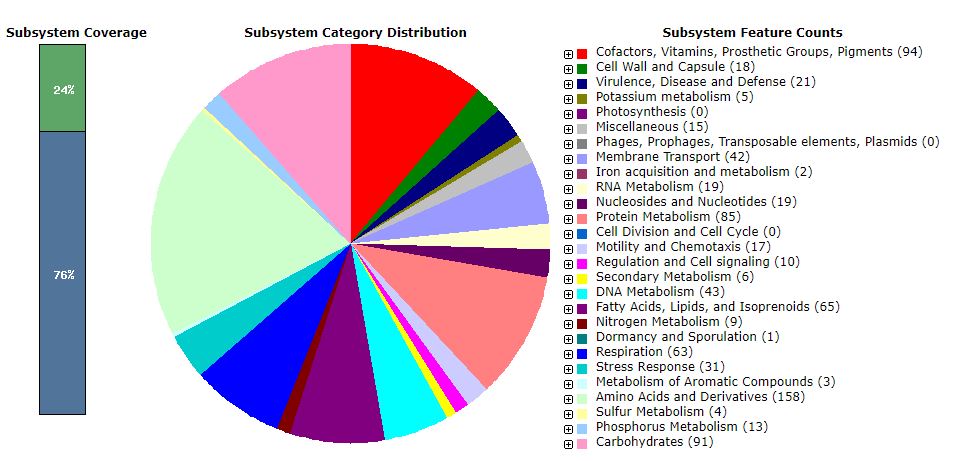
**

(c)

**Fig. S6**. RAST Subsystem category (https://rast.nmpdr.org) of the draft genome of strains (a), *Winogradskyella luteola*  WHY3^T^; (b)*, Erythrobacter ani* WH131^T^; (c), *Erythrobacter crassostrea* WH158^T^.

**Table S5.** RAST analysis result (https://rast.nmpdr.org) of the draft genome of strains 1, *Winogradskyella luteola* WHY3^T^; 2, *Erythrobacter ani* WH131^T^; 3, *Erythrobacter crassostrea* WH158^T^.

| **Subsystem Feature** | **1** | **2** | **3** |
| --- | --- | --- | --- |
| Cofactors, Vitamins, Prosthetic Groups, Pigments  Biotin  Cofactors, Vitamins, Prosthetic Groups, Pigments - no subcategory  Tetrapyrroles  Riboflavin, FMN, FAD  Pyridoxine  NAD and NADP  Folate and pterines  Coenzyme A  Quinone cofactors | 123  1  4  10  12  0  10  50  14  12 | 110  5  5  14  13  9  11  42  11  0 | 94  5  5  11  13  7  8  35  10  0 |
| Cell Wall and Capsule  Gram-Negative cell wall components  Cell Wall and Capsule - no subcategory  Capsular and extracellular polysacchrides  Gram-Positive cell wall components  Cell wall of Mycobacteria | 29  1  8  20  0  0 | 28  1  12  15  0  0 | 18  1  8  9  0  0 |
| Virulence, Disease, and Defense  Resistance to antibiotics and toxic compounds  Invasion and intracellular resistance | 24  12  12 | 34  22  12 | 21  13  8 |
| Potassium metabolism  Potassium metabolism - no subcategory | 9  9 | 6  6 | 5  5 |
| Photosynthesis  Light-harvesting complexes | 0  0 | 2  2 | 0  0 |
| Miscellaneous  Plant-Prokaryote DOE project  Miscellaneous - no subcategory | 10  9  1 | 19  15  4 | 15  14  1 |
| Phages, Prophages, Transposable elements, Plasmids  Phages, Prophages | 7  7 | 0  0 | 0  0 |
| Membrane Transport  Protein secretion system, Type II  Protein translocation across cytoplasmic membrane  Cation transporters  Uni- Sym- and Antiporters  Membrane Transport - no subcategory  TRAP transporters  Protein and nucleoprotein secretion system, Type IV | 27  0  2  4  2  19  0  0 | 100  15  3  6  2  22  0  52 | 42  10  4  1  2  23  2  0 |
| RNA Metabolism  RNA processing and modification  Transcription | 41  18  23 | 36  18  18 | 19  6  13 |
| Nucleosides and Nucleotides  Pyrimidines  Purines  Nucleosides and Nucleotides - no subcategory  Detoxification | 65  18  37  6  4 | 50  12  27  7  4 | 19  0  12  4  3 |
| Protein Metabolism  Protein folding  Selenoproteins  Protein biosynthesis  Protein processing and modification  Protein degradation | 139  5  0  102  14  18 | 164  9  1  129  7  18 | 85  8  1  56  6  14 |
| Cell Division and Cell Cycle | 3 | 0 | 0 |
| Motility and Chemotaxis  Flagellar motility in Prokaryota | 0  0 | 0  0 | 17  17 |
| Regulation and Cell signaling  Regulation and Cell signaling - no subcategory  Programmed Cell Death and Toxin-antitoxin Systems | 6  6  0 | 11  11  0 | 10  10  10 |
| Secondary Metabolism  Biosynthesis of phenylpropanoids  Plant Hormones  Secondary Metabolism - no subcategory  Plant Alkaloids | 5  0  4  0  1 | 5  1  4  0  0 | 6  0  4  2  0 |
| DNA Metabolism  DNA repair  CRISPs  DNA Metabolism - no subcategory  DNA replication  DNA uptake, competence | 53  40  2  5  4  2 | 67  48  0  9  8  2 | 43  31  0  3  7  2 |
| Fatty Acids, Lipids, and Isoprenoids  Isoprenoids  Phospholipids  Triacylglycerols  Fatty acids  Fatty Acids, Lipids, and Isoprenoids - no subcategory | 50  20  0  0  22  8 | 80  15  0  1  41  23 | 65  12  0  1  33  19 |
| Nitrogen Metabolism  Nitrogen Metabolism - no subcategory  Denitrification | 7  6  1 | 9  9  0 | 9  9  0 |
| Dormancy and Sporulation  Dormancy and Sporulation - no subcategory | 1  1 | 1  1 | 1  1 |
| Respiration  ATP synthases  Biotin  Electron accepting reactions  Electron donating reactions  Respiration - no subcategory | 19  8  1  1  2  8 | 83  10  5  8  35  30 | 63  0  5  7  34  22 |
| Stress Response  Osmotic stress  Oxidative stress  Detoxification  Stress Response - no subcategory  Periplasmic Stress | 20  4  9  4  2  5 | 51  3  35  6  8  3 | 31  3  16  5  7  3 |
| Metabolism of Aromatic Compounds  Peripheral pathways for catabolism of aromatic compounds  Metabolism of central aromatic intermediates  Metabolism of Aromatic Compounds - no subcategory | 9  1  7  1 | 8  2  5  1 | 3  1  2  0 |
| Amino Acids and Derivatives  Amino Acids and Derivatives - no subcategory  Glutamine, glutamate, aspartate, asparagine; ammonia assimilation  Histidine Metabolism  Arginine; urea cycle, polyamines  Lysine, threonine, methionine, and cysteine  Branched-chain amino acids  Aromatic amino acids and derivatives  Proline and 4-hydroxyproline  Alanine, serine, and glycine | 169  0  16  11  12  31  23  35  3  38 | 214  3  17  9  0  56  51  36  10  32 | 158  0  14  8  0  39  25  34  8  30 |
| Sulfur Metabolism  Sulfur Metabolism - no subcategory | 8  8 | 5  5 | 4  4 |
| Phosphorus Metabolism  Phosphorus Metabolism - no subcategory | 16  16 | 20  20 | 13  13 |
| Carbohydrates  Carbohydrates - no subcategory  Central carbohydrate metabolism  Di- and oligosaccharides  One-carbon Metabolism  Organic acids  Fermentation  Sugar alcohols  Monosaccharides  CO2 fixation  Polysaccharides | 93  1  45  3  4  0  15  8  11  2  4 | 136  1  68  9  4  5  39  5  5  0  0 | 91  1  39  3  5  5  29  0  9  0  0 |
| Iron acquisition and metabolism  Iron acquisition and metabolism - no subcategory | 1  1 | 0  0 | 2  2 |

**Tabel S6**. MIC values (%) of inhibition test with extracts from strains WHY3^T^, WH131^T^, and WH158^T^. Test strains were: 1, *E. coli* wild type BW25113; 2, *E. coli* acrB JW25113; 3, *P. aeruginosa* DSM 19882; 4, *S. aureus* Newman; 5, *C. freundii* DSM 30039; 6, *A. baumannii* DSM 30008; 7, *B. subtilis* DSM 10; 8, *M. smegmatis* ATCC 700084; 9, *M. hiemalis* DSM 2656; 10, *W. anomalus* DSM 6766; 11, *C. albicans* DSM 1665.

| **Microorganisms** | **1** | **2** | **3** | **4** | **5** | **6** | **7** | **8** | **9** | **10** | **11** |
| --- | --- | --- | --- | --- | --- | --- | --- | --- | --- | --- | --- |
| WHY3^T^ | - | - | - | 0.84 | - | - | 0.84 | 6.67 | - | 3.37 | 0.84 |
| WH131^T^ | - | - | - | - | - | - | - | - | - | - | - |
| WH158^T^ | - | - | - | - | - | - | 6.67 | - | - | 6.67 | - |

MIC value = 6.67- 3.34% (low activity); 1.67 - 0.42% (moderate activity); and 0.21 - 0.05% (strong activity).
